# Evaluation of the feasibility and sustainability of the joint human and animal vaccination and its integration to the public health system in the Danamadji health district, Chad

**DOI:** 10.1186/s12961-021-00688-z

**Published:** 2021-08-11

**Authors:** Mahamat Fayiz Abakar, Djimet Seli, Filippo Lechthaler, Lisa Crump, Arielle Mancus, Nhan Tran, Jakob Zinsstag, Daniel Cobos Muñoz

**Affiliations:** 1 Institut de Recherche en Elevage pour le Développement, P.O Box 433, N’Djamena, Chad; 2 Swiss Tropical and Public Health Institute (Swiss TPH), CH-4002 Basel, Switzerland; 3 University of Basel, Petersplatz 1, CH 4001 Basel, Switzerland; 4 Centre de Recherche en Anthropologie et Sciences Humaines, P.O. Box 6542, N’Djamena, Chad; 5 School of Agricultural, Forest and Food Sciences, Bern University of Applied Sciences, P.O. Box 3052, Zollikofen, Switzerland; 6 World Health Organization, Geneva, Switzerland

**Keywords:** Joint vaccination, Feasibility, Sustainability, Mobile pastoralists, One health

## Abstract

**Background:**

One Health approaches such as the Joint human and animal vaccination programmes (JHAVP) are shown to be feasible and to increase health care access to hard-to-reach communities such as mobile pastoralists. However, the financial sustainability and the integration into the public health systems at the district level of such programmes are still challenging. The main objective of the present study was to give insight to the feasibility and financial sustainability of JHAVP integrated as part of the public health system in Chad.

**Methods:**

We conducted a mixed methods study using semi-structured key informant interviews, focus group discussions and budget impact analysis. Strengths, weaknesses, opportunities, and threats were analysed regarding the feasibility and sustainability of the implementation of JHAVP in Danamadji health district in Chad. Feasibility was further analysed using three dimensions: acceptability, implementation, and adaptation. Financial sustainability of JHAVP was analysed through budget impact analysis of implementation of the programme at district level.

**Results:**

The acceptability of this approach was regularly assessed by immunization campaign teams through evaluation meetings which included pastoralists. The presence of authorities in the meetings and workshops of the programme had an incentive effect since they represent a mark of consideration these populations generally declared to be lacking. The coordination between the public health and veterinary services at central and decentralized level seemed to be a key element in the success of the implementation of the programme. Regarding financial sustainability, the total incremental budget impact was 27% slightly decreasing to 26% after five years, which accounts for up to one third of the total budget of the district health office. Also, given that most of the costs for each round are recurrent costs, efficiency gains from scale effects over time are limited.

**Conclusion:**

Based on these findings, we conclude that for JHAVP to be routinely delivered at the district health level, a considerable increase in financial resources would be required. The district could benefit from joint immunization to maintain contact with mobile pastoralists to promote the use of available immunization services at district level.

## Background

Mobile pastoralist communities in the Sahel region, are largely excluded from social and health services and rarely considered as beneficiaries of national health and development interventions because of their mobile lifestyle that take them across multiple countries in some cases (1–3). Earlier studies in rural areas of Chad showed high frequencies of fever-related illness, anaemia, respiratory and gastro-intestinal illnesses [4–6]. Antenatal care service utilisation was systematically lower for mobile pastoralists when compared to rural settled populations [7]. Quality of health services is perceived as poor by nomadic communities [8], mainly because of the lack of national policies adapted to their context. Even in countries that have health policies specific for mobile pastoralist communities, these policies often neglect addressing essential elements such as gender disparities, high maternal mortality rates or child health care [9]. Unpublished data from a survey among mobile pastoralists conducted in May 2015 show that Polio 1 and Polio 3 coverage were 11.6 and 2.7% respectively and Penta1 coverage was 0% among pastoralist communities in the Danamadji health district in Chad, which is considerably lower than for the sedentary populations [10].

There are examples of interventions tailored to meet the need of mobile pastoralist communities through the collaboration between human and animal health sectors coined “One Health” [11]. “One Health” can be defined as the added value in terms of improved human and animal health or financial savings or environmental services resulting from a closer cooperation of both health sectors [12, 13]. Joint human and animal vaccination programmes (JHAVP) in Chad have shown to be effective in providing health care access to previously inaccessible communities and save resources through sharing transport, equipment and logistics [14].

To strengthen the JHAVP among nomadic populations, the strategy was to develop a holistic programme of integrated health activities. This programme goes beyond the health aspects to include education, safety, pastoral wells and so on. A milestone was reached with the creation of the Governmental Nomadic and Islander Community Health programme (“*Programme National de Santé des Nomades, Insulaires et zones d’accès difficile*” (PNSN): Decision No. 227 MSPASSN/SE/SG/DGRP/2014 [9]. Until recently, the joint human and animal vaccinations of the nomadic populations were the result of initiatives of NGOs, without a real institutional body, untill the establishment PNSN in 2014.

JHAVP were usually implemented as part of vertical interventions with most of the financial support coming from donors with little experience on the integration of this approach in the routine health system of the country. In this sense, there is a need to better understand whether cost-effective interventions also translate into affordability, considering especially the low health budget allocations in low- and middle-income countries [15, 16]. The integration of these joint programmes as a routine activity into existing infrastructure and programmes funded by the district health system could require substantial financial reallocations putting a strain on the delivery of other essential health services. The benefits of the integration of vertical interventions in local health systems such as primary health care are well documented and vaccination programmes are one example of that [17]. Although there is some debate about the definition and scope of integration of health services [18], there is a substantial body of knowledge supporting the integration of services to improve health system performance [19].

However, the operational arrangements and users’ perspectives on the integration of vertical interventions are not yet well understood. The objective of the present was to give insight to the feasibility and financial sustainability of joint human and animal vaccination programmes integrated as part of the local health system in the Danamadji health district in Chad. The present study is part of a PhD thesis published in 2017 [20].

## Methods

### Study setting

Our study took place in the Danamdji health district (province of Moyen-Chari), in the southern part of Chad bordering the Central African Republic, one of three health districts of this Region. Its population is 128,369 inhabitants [21] including mobile pastoralists mainly from Arabs and Fulani cattle breeders. The district has 18 functional health zones, 17 health centres and 1 district hospital. According to a recent household survey, health service utilization, especially vaccination, is significantly lower among nomad communities than in sedentary populations.^1^

### Qualitative methods

#### Data collection methods

Researchers experienced in qualitative methods were recruited and supervised by IRED^2^ and CRASH^3^ to conduct key informant interviews (KII) and facilitate focus group discussion (FGD) among relevant stakeholders involved in the design, implementation, or management of JHAVP. KIIs and FGD were conducted in French or in Arabic, and subsequently recorded, transcribed, and translated into French.

Semi-structured KIIs were conducted with 10 participants from public and veterinary health authorities in the province of Moyen-Chari and the Danamadji health district involved in the programmes and representatives of the nomadic communities.

We also conducted a participatory analysis following the Strength, Weaknesses, Opportunities and Threats (SWOT) analysis among the same 10 participants gathered in a two days’ workshop (see Table 1).

**Table 1:** Study participants (KII and FGD-SWOT)

Target population	Number of KII
Public health delegate for the Moyen-Chari region	1
Livestock delegate for the Moyen-Chari region	1
Medical chief of Danamadji health district	1
Research coordinator at CSSI^a^	1
Chief of health zone responsibility of Danamadji	1
Chief of veterinary post of Danamadji	1
Chief of veterinary post of Roro	1
Responsible of the expanded immunization programme, Province of Moyen-Chari	1
Nomads representative	1
Responsible of the national programme of health of nomads PNSN^b^	1
**Total**	10

^a^Centre de Support en Santé International (CSSI); ^b^Programme National de Santé de Nomades (PNSN)

The workshop participants were chosen because of their involvement in the life of nomads in general or as the health-related representatives of local community’ organizations and based on their knowledge of the pastoral environment and the delivery of health services to the target populations of the study. In addition, stakeholders involved in the implementation of JHAVP at central and decentralized levels were invited. Thus, these participants were deemed worthy of investigation because each, by their position, have a piece of knowledge on the issue of health of mobile pastoralists and/or their livestock.

The participants were selected based on the list of the stakeholders involved in the last two campaigns of JHAVP. The nomadic representative was appointed by a local nomads’ organisation to participate in the workshop as he speaks French in addition to Chadian Arabic.

#### Data analysis

We used the framework develop by Bowen et al. [22], complemented with the framework proposed by Schell et al. [23] in our analysis to assess the feasibility of the JHAVP. A SWOT analysis identified strengths, weaknesses, opportunities, and threats regarding the feasibility and sustainability of JHAVP [24–26].

Bowen et al. recognized that there are no ready-made criteria to understand the feasibility of health programmes. They define a number of areas which have been the focus of studies looking at the feasibility of programmes [22]. The key questions that were relevant for the continuation or expansion of the JHAVP were related to whether the approach was accepted by the different actors involved in the programme and to what extent the intervention can be integrated in the local health system and move from an externally controlled environment to an uncontrolled one. The team decided to focus on three of the eight areas (acceptability, implementation, and adaptation) as described by Bowen et al.

First, we explored the JHAVP acceptance among nomadic communities and whether it is accepted voluntarily or not. Second, we were interested in the implementation of the programme among nomads who live in an environment dominated by a mobile lifestyle and limited access to basic social services. Specifically, we explored whether the implementation strategy of the JHAVP, from a logistic and operational point of view, took these realities into account to meet the needs of nomads. Finally, we investigated whether the programme was designed considering the social norms and perceptions of the nomadic communities of Danamadji district.

We draw information from the KIIs, the FGD and the specific participatory SWOT workshop to populate the SWOT matrix. Key messages were identified and then coded into themes. The SWOT analysis session provided complementary data to the KII information which allowed more precise conclusions regarding the feasibility and sustainability of the JHAVP.

### Budget impact analysis

#### Data collection

Data on resource use and local prices were gathered from the health district accounting system in Danamadji as well as from reports that resulted from a mixed vaccination campaign held in 2016 in the same district. Taking the perspective of the district health system, the purchase costs of human vaccines were not included as these costs are typically borne by the Expanded Programme on Immunization (EPI). Meanwhile, livestock vaccines purchase costs were covered through direct payment by animal owners on a cost recovery basis. Furthermore, all costs related to higher functional levels (province, nation) such as expenditures for administrational and managerial support as well as costs assigned to the veterinary sector (i.e., livestock vaccines) were not considered for the Budget impact analysis (BIA). Base salaries for field personnel were not included (only allowances) as this expenditure is typically handled at the national level and it was assumed that the campaign would draw on already salaried health workers in the district.

#### Data analysis

The design of the BIA approach considers financial costs building on a costing model that describes the implementation of a JHAVP (one outreach event) and which is based on detailed data from local accounting systems. Reporting of the BIA follows established guides [27].

An overview of the basic assumptions and most essential elements of the study design is detailed in Table 2. The analysis takes the perspective of the human health-care provider at district level using the case of Danamadji. The target population of JHAVP were children less than 60 months of age who are part of hard-to-reach communities, typically mobile pastoralists. As the size of pastoralist communities was largely unknown, we derived the initial total population size of mobile pastoralists present in Danamadji based on the number of children reached through a joint vaccination campaign supported by Swiss TPH and implemented in 2016 in the same district (*n* = 1684).

**Table 2:** BIA design and assumptions

	Design and assumptions
Perspective	Human health care provider at local level (health district): costs borne at higher levels or related to the veterinary sector were not taken into account
Time horizon	5 years, capital costs were not annualized
Target population	Children from mobile pastoralist communities in the health district < 60 months (= 1750)
Initial population	Estimation based on the number of nomadic children reached during a mixed vaccination campaign in 2016 assuming that the intervention covered the total population at that time
Population growth rate	3%
Cost of the intervention	Derived from project reports and accounting system of a mixed vaccination campaign implemented in Danamadji in November 2016
Current cost of interventions aimed at reaching mobile pastoralist communities for vaccination	Since there is no specific intervention to reach remote populations, we assumed that no resources are currently used
Economic impact	Economic consequences stemming from improved health outcomes through increased vaccination coverage among mobile pastoralists are not taken into account
Health district expenses	Derived from the official accounting system 2016

Assuming that most children less than 60 months of age from pastoralist communities in the district have been attended to during the campaign, we derived the total initial population in the district applying the proportion of children below 60 months derived from routine statistical indicators for sedentary populations living in the district (16.5%).

According to a health service utilization status in the study district [28], vaccination coverage among mobile pastoralists was very low in 2016 (around 1%). We therefore assume that there is currently no employment of resources (and no costs) for delivering vaccination services to mobile pastoralists. This implies that the total budget impact of implementing a combined vaccination programme corresponds to the incremental budget impact.

Possible financial consequences stemming from improved health outcomes through increased vaccination coverage among mobile pastoralists were not considered. Building on a provider perspective, private costs incurred by households to participate in the vaccination campaign were not included. A five year’ time horizon was chosen to examine possible scale effects over time.

### Ethical considerations

The study received ethical clearance from the Nation bioethics committee in Chad (“*Comité National de Bioéthique du Tchad*”: Décision N°186/PR/PM/MESRS/SG/CNBT/2016, and the WHO Research Ethics Committee Review (WHO ERC): 12/04/2016, Protocol ID: ERC.0002684, 18/03/2016). Interview sessions always began with reading the survey information sheet and asking the respondent if he/she has any questions before signing the consent form. Participants in the survey were given the choice to decide whether interviews should be recorded or not.

Respondents were free to interrupt or stop interviews at any time or choose not to answer some specific questions. The confidentiality of the interview and the anonymity of the respondent were assured. Interviews took about 30–45 min. They were held in Sarh city, capital of the province of Moyen-Chari during a workshop where all targeted people were invited.

## Results

We conducted a mixed methods study with qualitative and quantitative methods including 10 semi-structured KIIs, a FGD during the SWOT analysis, and a budget impact analysis of the implementation of JHAVP campaigns based on the health district accounting system and the previous JHAVP reports.

The main questions asked were:

- What are the main strengths and weaknesses of the JHAVP, how was it coordinated, what are the provided services and was it adapted to the nomads’ context?
- What are the main lessons learned from the JHAVP and how was it perceived by the local population?
- What are the main financial and implementation challenges facing the JHAVP and does it have a future?

The feasibility of the programme was analysed with regard to acceptability, implementation and adaptation to the local context [22].

### Acceptability

The acceptability of this approach has been the subject of on-going assessments by immunization campaign teams. Meetings were regularly held to evaluate the JHAVP activities to which the representatives of the nomads were invited.

"Indeed, during the implementation of the JHAVP, we did an evaluation meeting of the activities we had to organize and the reaction of this community is that this kind of activities should be repeated more often, because they think it’s good to bring an additional package of services that is often goes beyond vaccination". (Head of an NGO).

This tendency among nomads to prefer the JHAVP was confirmed by a regional health official in the province of Moyen-Chari.

"I think this programme was much appreciated by the nomads, because after the activities we tried to hold meetings with the various actors to determine the bottleneck that prevents the children of the nomads to come to vaccination. And they (nomads) have spoken in favour of joint vaccination which is an opportunity for them to benefit from its activities."

Unlike other vaccination campaigns (outreach strategy and routine immunization), the JHAVP starts with an official gathering where high authorities, the Minister in charge of Public Health and the Ministry in charge of Livestock, participates and advocate for vaccination. The presence of these authorities provides an incentive effect since they represent a mark of consideration which the nomadic populations generally find lacking.

The acceptance of the JHAVP by nomadic populations was confirmed by officials and nomads’ representatives.

"The joint vaccination approach has paid off, as long as it has mobilized resources. Seeing the results, we have never reached this coverage level in our routine immunization activities. I do not have the number in mind, but the approach has allowed us to reach nomadic children who have never been vaccinated since they were born until the age of five ". (Delegate, province of Moyen-Chari).

A representative of the nomads participating in the workshop agreed in the same direction confirming that:

"On the side of the nomads where I am the representative, everyone is on the same wavelength as this joint vaccination operation is beneficial and everyone wants it to happen every year."

### Implementation

The coordination between the public health and veterinary services at central and decentralized level was found to be a key element in the success of the implementation of the JHAVP.

"There are many consultations between the two ministries during the implementation of this approach. I wanted to say that when it comes to a disease that is common between humans and animals, the two ministries always meet to think about the strategies to adopt". (Delegate, province of Moyen-Chari).

The strategy of organising the vaccination campaigns for humans and animals combined into one single activity in a central place brought positive effects according to nomadic communities. This gives the nomads the opportunity to interact with other communities and to trade goods which was an efficient way to motivate these communities to participate in such activities. This was noted by a health service worker who took part in the JHAVP held in 2013 in the Danamadji health district.

"It was a great joy, a great reunion. Breeders who have separated from each other for a long time have found themselves together again. (….), nomads often like these kinds of opportunities because it allowed them to access health services to their children, their pregnant women (….)".

### Adaptation

Involving social mobilization teams from within the nomadic communities is one of the strategies used to adapt the JHAVP to the socio-cultural and health context of these communities. The report of the last JHAVP stated that a total of 36 social mobilizers were identified among nomads’ representatives and trained in the Danamdji and Kyabe health districts.

It is well-known that one of the basic characteristics of nomadic communities in the Danamadji health district is the low access to health services in general and immunization in particular [10]. Depending on the severity of the conditions, nomads would consider attending public health facilities to seek care. However, as reported by Abakar et al. [ref] they were discouraged either by the cost of care, or the possibility of facing discrimination [8]. Therefore, providing joint human and animal health services in an additional package of health services beyond vaccination is a mean to adapt the JHAVP to the specific needs of nomads.

"We intervened by bringing a joint package of human and animal vaccination and taking care of the mothers at the camps level. Our teams have nurses within them to make rapid consultations for the sick people. Also, our teams have some drug supply to take care of the minor health problems". (An NGO representative).

This is also confirmed by a delegate from the province of Moyen Chari who adds:

"The joint vaccination strategy is not just about vaccination. We take advantage of this approach to do primary prevention against malaria, deworming of nomadic children and vitamin A supplementation. We also do pre-natal consultation. In short, we were able to reach these communities with activities that, without this approach, would be difficult to achieve".

### SWOT analysis

Table 3 summarizes the main findings of the SWOT analysis realized during our study.

**Table 3:** SWOT analysis summary

	Opportunities Political stability Political good willingness with regard to JHAVP Funders adherence to the approach	Threats Security Scarcity of funds Natural catastrophes Breeders agricultures conflict
Strengths Existence of framework Availability of personnel Coordination at all levels	SO strategies Establishment of inter-sectoral dialogue framework Holding donors meetings for mobilization of additional funding	ST strategies Establishment of inter-sectoral platform for collecting and analysing information needed to anticipate potential threats Promotion of dialogue between communities (sedentary and nomads)
Weaknesses Absence of legal basis Insufficient funds Absence of additional health intervention (rabies, CBPP, etc.)	WO strategies Establishment of inter-ministerial entity for the implementation of JHAVP Advocacy for resources mobilization	WT strategies Establishment of a legal basis to the JHAVP Advocacy for resources mobilization

#### Strengths

Although JHAVP is implemented informally (it does not yet have a formal institutional framework that can guarantee its sustainability), it still has strengths that allow it to survive and continue to provide a number of services to nomadic populations and their livestock.

##### Existence of financial support from donors

One of the conditions for the sustainability of any health programme, such as the JHAVP, is the availability of financial resources. Indeed, such a health programme requires substantial financial resources. "*Yes, there is funding*", a delegate from the province of Moyen-Chari said. The delegate further added "*When there is a strong political will, it means that the finances will follow as well*".

Even though there is not yet a substantial governmental funding for the programme because a special service for this purpose is still lacking, there are some NGOs working in this field for more than a decade. Additionnaly, other NGOs and UN agencies can provide financial support for the JHAVP, although this may not be enough to ensure its regularity.

"(…). Because I know that there is now in Moyen-Chari, many projects like the Programme d’appui aux districts sanitaires au Tchad (PADS), there is also the project led by IRED (AHPSR) and the health project of the mobile pastoralists in Central Africa lodges at the CSSI. There is also the MSF who is intervening". (An NGO representative).

###### Existence of a reference framework document

The existence of a reference framework document^4^ adopted by the Government of Chad to define inter-sectoral support programmes to nomadic communities was considered as strength. Although the various objectives and recommendations contained in this document are not yet translated into practice, this document constitutes a reference framework to which the various actors working in support of nomadic populations can refer for the implementation of health approaches and other integrated activities for the benefit of nomadic populations and their livestock.

###### Existence of the programme of health of nomads (PNSN)

This institution is created to serve as a framework for reflection, orientation, and planning of health activities for nomadic populations and, in a broad sense, hard to reach populations.

"Well, if I have one last thing to add, it may be a suggestion. It is to advocate with the Ministry of Health so that all actors involved in the nomadic health sector can get around this programme, work in synergy. The interventions of certain partners, notably the NGOs, must not be allowed to escape the national coordination of the PNSN, which is today the nomadic health programme which is for us a key partner and which already shows the good will of the government to appropriate the thing. (…)" (An NGO representative).

###### Availability of qualified vaccinators and supervisors

Indeed, although the logistics and per diem was always financed by NGOs, the management of these vaccines is largely the responsibility of the health and veterinary officers. Apart from the few people recruited as community health workers responsible mainly for communication, most vaccinating agents are qualified government personnel.

"At the level of the health districts, there is the District Medical Officer (DMO) who coordinates activities at the district level. When we go out here and we go for the vaccination, he knows that in such a place it is such person who goes to vaccinate. There is an implementation plan. At the regional level, the delegate coordinates. I remember when I was in Danamadji, it was the DMO who was in charge of coordinating our activities at Danamadji level. "

#### Weaknesses

Although its regularity is not assured (since its first implementation in 2000, it has only been executed two or three times), the JHAVP benefited from factors which guaranteed its survival. However, it has some weaknesses such as lack of a proper institutional framework, insufficient financial support, lack of implementation infrastructure and lack of socio-anthropological study among nomads to improve the performance of this approach.

#### The non-institutionalization of JHAVP

Although JHAVP seems to be well appreciated by nomadic populations, this initiative suffers from several disabilities which could undermine its regularity and sustainability. One of the weaknesses is the non-institutionalization of the programme. Indeed, the programme is a transverse health operation between the Ministry of Health and the Ministry of Livestock, but there is a lack of a transverse institution with a legal basis capable of managing the integrated health of nomads and their livestock, as a regional delegate rightly observed:

"There is no proper framework for managing this integrated human and animal health operation. Until then, now the things worked based on the good relationships between the human and animal health authorities that we are".

This concern is widely shared by the relevant service officers in, human health and animal health, such as a regional delegate who offered this suggestion:

"And here I think it is necessary to think about creating a formal framework of consultation in order to manage this issue of human health and animal health, for example an inter-ministerial decree".

#### Insufficient financial resources

As there is not yet an institutional service within the districts implementing JHAVP, there are not sufficient financial resources from the government. Most of the operational costs are covered by external donor’ contributions.

"Now, the weaknesses are insufficient funding especially from the government. It is an activity that should be consistently applied, because interrupting it for a year or two is a handicap". (Provincial delegate, province of Moyen-Chari).

#### Construction of fences for livestock vaccination is a bottleneck

The nomadic populations in Chad including those in Danamadji are very invested in their livestock, as confirmed by a vaccination programme officer in the region:

"Yes indeed, the statement of nomadic communities better vaccinates their cattle than their children are true. This can be justified by the absence of nomads in the immunization service. Everywhere in the health centres, we do not notice the presence of the nomads."

Therefore, the success of JHAVP depends on the success of animal vaccination, as one NGO official states. Animal vaccination is set up as a gate of entrance to the nomadic populations who are looking for any initiative for the health of their livestock. Thus, it was thought that by offering them the opportunity to come to vaccinate their cattle, human health workers could vaccinate their children. Meanwhile, the success of animal vaccination depends on the availability of vaccination fence (park or enclosure) which creates some problems:

"Among the challenges, there is the question of the enclosure (…). You know at the bush there, if you want to make an enclosure with the woods, you will have problems with the agents of waters and forests. Not long ago, the Livestock Delegate told me to do an enclosure and as soon as it was done with the woods, we had problems with water and forest agents. (…) and I ended up paying something to solve problem". (A nomads’ representative).

### Budget impact analysis (BIA)

The main budget characteristics of the mixed campaign are represented in Table 4 and Table 5 in USD (1 USD = 616 FCFA, January 2017). Table 4 presents resource consumption in natural units and the corresponding unit costs for a mixed vaccination campaign realized in 2016 in the study district. The total cost of the outreach event was 17′328 USD with 1684 children vaccinated. Table 5 shows an overview of the main cost categories: transportation is the budget category with the greatest weight followed by personnel and logistics (e.g., basic equipment including chairs, tents, and refreshments for participants). Considering costs incurred only at the district level (excluding personnel costs at regional and national level), the total cost was 14′384 USD.

**Table 4:** Composition of the main cost categories for a mixed vaccination campaign realized in 2016 in the study district

	Unit	Quantity	Unit price	Total cost (% fixed)
Transportation				
Fuel	Litres	1750	0.97	1697.5 (0)
Car rentals	Days	52	121.75	6331 (0)
Logistics				
Social mobilisation	Lumpsum	NA	NA	487 (1)
Tents	Day*quantity	20	8.1	162 (0)
Chairs	Days*quantity	200	0.24	48 (0)
Mats	Lumpsum	NA	NA	146 (1)
Blankets	Lumpsum	NA	NA	156 (1)
Public adress system	Lumpsum	NA	NA	81 (1)
Refreshments	Drinks	1684	0.2	336.8 (0)
Personnel/administration				
Supervisor national program	Person days	91	49	4459 (0)
Supervisor regional program	Person days	20	24	480 (0)
Supervisor district	Person days	20	16	320 (0)
Midwife	Person days	20	16	320 (0)
Community worker	Person days	40	8	320 (0)
Human vaccinator	Person days	40	10	400 (0)
Animal vaccinator	Person days	40	10	400 (0)
Recorder	Person days	40	8	320 (0)
Drivers	Person days	36	24	864 (0)
Total costs				17′328.3

The “social mobilisation” item includes costs for organising communication material including local radio spots, announcements on papers, and illustrative pictures. The remaining items under the logistics category cover costs generated by the campaign operation

**Table 5:** Cost characteristics of a joint vaccination campaign in Danamadji

	Total costs (USD)	Total costs district (USD)	Share public health sector (%)	% fixed	Average costs per child (public health sector, USD)	Marginal costs per child(public health sector, USD)
Transportation	8028.5	8028.5	50	0	2.38	2.38
Logistics	1416.8	1416.8	50	57	0.42	0.16
Personnel	7883	2944	79	0	1.28	1.28
*Total*	17′328.3	14′384.3			5.55	5.29

The allocation of the costs of resources used to the veterinary and public health sector is based on equally divided shares for the transportation and the logistics category assuming comparable utilization of these basic inputs. Personnel costs were distributed proportionally according to health workers present during the campaign, with 79% being allocated to the public health sector. The share of fixed costs was rather low and only applicable to costs related to logistics (57%), which is the budget category with the lowest share in total costs (Table 5). Accordingly, it can be noted that, apart from the logistics category, marginal costs correspond to average costs which indicates little room for economies of scale. Average cost per vaccinated child (without costs of vaccines) was around 5.50 USD.

Table 6 shows the budget impact of realizing one intervention over a one-year time horizon. The district target population was calculated based on the demographic parameters presented in Table 2. Incremental costs were computed by extrapolation, multiplying the marginal cost per child for each cost category (Table 5) with the number of the target population while fixed costs were held constant. The total incremental budget impact was 27% meaning that the realization of one JHAV campaign would use up almost one third of the district’s allocated funds. Assigning the different types of expenditures to their corresponding budget category shows that the burden is especially high with respect to human resources, where costs for personnel exceed the respective budget line by almost half (153%).

**Table 6:** Incremental budget impact of combined vaccination campaigns for the public health sector at district level

	Incremental costs at district level (USD)	Incremental budget impact (%)	Incremental budget impact per budget category (%)
Transportation	4172.6	15	23
Logistics	1154.1	4	51
Personnel	2236.3	8	153
*Total*	7562.0	27	

**Fig. 1:**
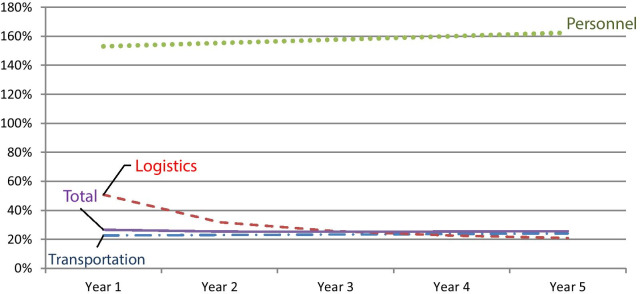
Incremental budget impact for the public health sector at district level over a 5 years’ time horizon

Figure 1 examines the financial consequences over time where a combined vaccination campaign is conducted every year and assuming a constant population growth rate of 3%. More specifically, in Table 6, incremental costs for each year are computed by multiplying the marginal costs with the number of the target population assuming a yearly demographic increase of 3% while fixed costs are held constant. Figure 1 shows that the budget impact of logistic expenditures decreases considerably over time due to the high share of fixed costs as inputs can be reused over the course of time. However, as shown in Table 5, transportation and personnel expenditures are fully variable and therefore increase over time due to higher workloads based on an increasing target population. In total, the impact of the intervention on the district budget decreases slightly from 27 to 26%. Thus, with the share of fixed costs being relatively low, efficiency gains from scale effects over time are limited.

**Fig. 2:**
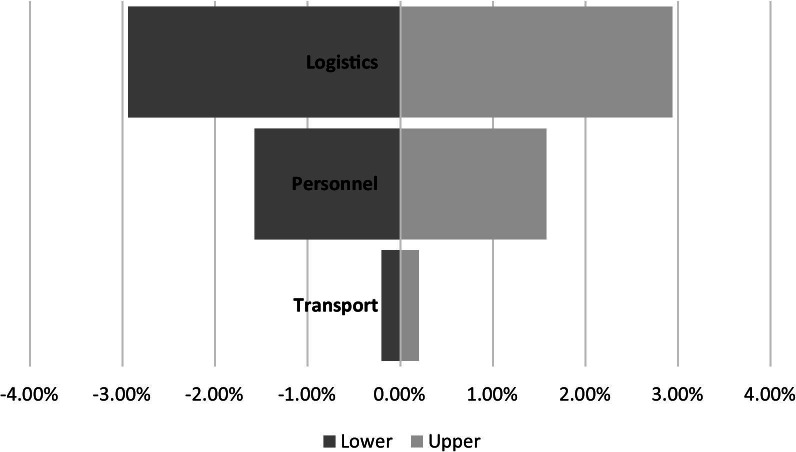
Deviation from baseline value of budget impact (27%) due to 20% deviation in the cost categories (in % points)

Deterministic sensitivity analysis was applied to assess uncertainty in key parameters. The upper and lower bounds were defined based on 20% deviation from the baseline value in marginal costs for transportation, logistics and personnel. The tornado diagram (Fig. 2) shows that incremental overall budget impact varies between 3% for variations in transportation costs and 0.2% for variations in logistic cost.

## Discussion

We conducted a mixed method study to assess the feasibility and sustainability of JHAVP integrated in the local health system in Danamadji health district in Chad.

Our results show that the joint delivery of human and animal health services, including joint immunization, is well appreciated by nomadic communities. The tendency among nomads to prefer joint vaccination was confirmed by several interviewees in our study. The magnitude of the event, marked by a large-scale official launch, played an important role in the acceptability and awareness of immunization in the nomadic community in Danamadji. The presence of the authorities during these events had an incentive effect since they represented a mark of consideration which the nomadic populations generally declared to be lacking.

The implementation of JHAVP was a great success mainly because of ongoing coordination and exchanges between the public health and livestock sectors. This resulted in several strategies to increase the success rate of implementation. Among these strategies is the organization of preparatory missions composed of the agents of two ministries for the mobilization as well as the involvement of the administrative, religious, and traditional authorities as facilitators. Also, the way the vaccination activities were implemented using a central location where all communities would come to vaccinate cattle and children not only facilitated the operations but also mobilized the media in the region.

The lack of trust towards local health systems is one of the factors limiting access to vaccination among nomads [8]. Another factor was using social mobilizers from nomads themselves and offering, besides vaccination, an additional package of health services such as prenatal consultations, distribution of impregnated bed-nets, vitamin A supplementation and many others (Report of the Joint Immunization Campaign, 2013).

According to Schelling and colleagues [29], in addition to sensitization on vaccination in these campaigns, nomads appreciated the quality and potential of health services and began to trust health service providers.

The sustainability of JHAVP has several strengths, including the existence of a document framework "*Programme d’appui intersectoriel aux communautés nomades au Tchad*" and the existence of the nomads’ health programme (PNSN) in the ministry in charge of public health. Added to this the current willingness of some donors to support this vaccination strategy to reach nomads.

It should be noted that all joint immunization campaigns organized in the past were supported by partners and implemented by central structures. This led us to examine sustainability from the point of view of the integration into the local health system at the district level through a budget impact analysis. Considering the affordability of the intervention at the district level through the lens of a budget impact analysis, this study shows that the financial burden on the local health system would be relatively high, comprising around one third of the health district budget.

This impact would be even higher considering that several outreach events are necessary to reach full immunization. With no external funds available, the implementation of a yearly campaign would use up around one third of the district’s health budget. This implies substantial consequences regarding budget reallocation which applies particularly to the most constrained budget categories such as human resources.

As the current BIA focused mainly on financial cost, possible opportunity costs of the health staff employed in the JHAVP was not quantitatively considered in this model. It is well known that health districts in sub-Saharan Africa are understaffed, which is also the case for the current study districts [7], implying that the integration of the intervention would require additional human resources.

As the perspective of the current study was limited to the human health care provider, the cost incurred at household level to access the service was not explicitly included. From a parallel study which was implemented in the same health district [7] it was calculated that willingness and ability to pay together with time spent for accessing the health services reduces health care coverage by around 25% for mobile populations. This indicates the need to consider private household costs to effectively implement JHAVP.

Joint vaccination campaigns, being an outreach activity, do not rely on substantial infrastructural investments such as buildings and fixed equipment. Interpreted from a costing perspective, this implies only minor shares of fixed cost with little room for economies of scale. Consequently, increasing the target population through geographical expansion of the intervention to other districts will only bring minimal efficiency gains.

In fact, the lack of information is one of the principle demand-side barriers for vaccination among these communities, whereas geographical barriers do not appear to be a major concern [8].

A current representative survey in the Danamadji district showed that 57% of mobile pastoralist households own a mobile phone [10]. Regular communication and exchange of vaccination-related information with the pastoralist communities in between the sporadically occurring campaigns could be managed in a less cost intensive way through systematic application of mobile phone technology.

Building on these findings, we conclude that for mixed campaigns to be delivered as a part of the routine district health system, a considerable increase in financial resources would be required. This can only be assured through continued collaboration with public and public–private partnerships such as the Expanded Programme on Immunization. If external funding is available, local health systems could continue to implement mixed campaigns to establish contact between nomadic communities and the health district to increase vaccination coverage. However, since the approach does not appear to be financially feasible at the local level over the long run, health districts need to enable less cost-intensive regular communication and information activities aimed at enhancing service utilization of vaccination services at the health facilities.

## Conclusion

Joint human and animal vaccination programmes among mobile pastoralist’ communities is operationally feasible in the Danamadji health district, if external funding is secured for this activity. However, while feasibility is not problematic, its sustainability raises concerns through some weakness and threats to this health programme. These weaknesses, which influence the regularity of the programme, could constitute serious long-term handicaps for its survival.

The integration of JHAW as a routine activity at the district level depends on the mobilization of additional financial resources, representing a considerable challenge to its feasibility. The district should therefore aim at maintaining contact with nomad communities through community work and regular communication to promote the less cost-intensive use of available immunization services at district level.

## Data Availability

The datasets used and/or analysed during the current study are available from the corresponding author upon substantiated request.

## Abbreviations

BIA: Budget impact analysis
CNBT: Comité national de bioéthique du Tchad
CRASH: Centre de recherché en anthropologie et sciences humaines
DMO: District medical officer
EPI: Expanded programme of immunization
ERC: Ethics review committee
FGD: Focus group discussion
JHAVP: Joint human and animal vaccination programmes
IRED: Institut de recherche en élevage pour le développement
KII: Key informant interviews
NGO: Non-governmental organization
PADS: Programme d’appui aux districts sanitaires au Tchad
PNSN: Programme National de Santé des Nomades, Insulaires et zones d’accès difficile
Swiss TPH: Swiss tropical and public health institute
SWOT: Strengths, weaknesses, opportunities and threats
WHO: World health organization

## Footnotes

1 For example, Polio 1 coverage was 11.6% among nomads compared to 80% among local sedentary populations.

2 Institut de Recherche en Élevage pour le Développement (IRED).

3 Centre de Recherche en Anthropologie et Sciences Humaines (CRASH).

4 Programme d’Appui Intersectoriel aux communautés nomades en République du Tchad.
